# The Neuroses of the Menopause

**Published:** 1897-05-15

**Authors:** W. H. C. Neunham

**Affiliations:** Physician Accoucheur Bristol General Hospital


### THE NEUROSES OF THE MENOPAUSE.

By W. H. C. Newnham, M.D.Cantab., Physician Accoucheur Bristol General Hospital.

The menopause, or climacteric, may be said to include all of that period of time intervening between the beginning of irregularity of menstruation and the complete cessation of the flow, with the subsequent restoration to health. Very few women pass the climacteric period without suffering more or less, and in some cases permanent damage is met with. At this time the vague nervous symptoms which accompany the disturbances incident to the change of life are ushered in. Where these exist, depending upon the approaching menopause, they must be included in this period. Very often, during this time, different symptoms are produced in different women. These symptoms include manifestations or perversions, especially of the nervous system, and are shown in the form of vertigo, faintness, flushes, cold hands and feet, loss of memory, irritability of temper, fear, apprehension, melancholia, and hysteria.

An awakening of sexual desire quite unknown in former years, which is often looked upon with a sense of shame, is not uncommon in women undergoing the menopause. These symptoms enumerated above are not to be met with in every woman at the menopause; they include the principal disturbances from a neurotic point of view observed at this time in a large number of women. The neuroses seem to be most commonly encountered, and the one symptom, that seems to be almost universally experienced at this time, is flushes. These nervous symptoms may be so severe as to result in mental derangement, and this may take the form of incurable dementia. Perhaps the most common and the most terrible form of mental disease developed at the climacteric is a tendency to the abuse of alcohol. It must be remembered in these cases that there is nearly always a strong inducement to the indulgence. Women are always secret drinkers, in this differing very greatly from men; a woman knows how much more she has to lose, and how much more misery and suffering she is likely to bring upon others. The cause will generally be found to exist in some physical suffering, or in some mental distress from which she seeks relief. In these cases it is always well to examine for some disease of the appendages; the removal of the offending portions has often proved a cure for these terrible manifestations of the climacteric. Another essential treatment is removal of the patient from all the former associations. At this time also the increased deposit of fat in the omentum frequently leads these patients to imagine they are either pregnant or suffering from some form of tumour or dropsy; and it is frequently most difficult to convince them and even their medical attendants that it is simply all "fat," and that time will really arrange it all. Some of these cases have without doubt been operated upon for supposed abdominal tumour. If the cessation of the menstrual flow is sudden, the deposit of fat in the omentum is occasionally enormous. The cause of these various neuroses would appear to be in the sudden congestions of certain areas of the nervous system, due to the non-escape of the customary monthly bloody discharge. Hæmorrhage from the mucous membrane of the nose, hæmorrhoids, free diarrhœa, or a profuse leucorrhea will often prove a relief to the above symptoms.

If the menopause should be attended by peculiar nerve-storms, then the physician should always be on the look-out for some pathological condition. This is the time in a woman's life when malignant disease of the uterus may show itself. When once the menopause is established, then there should never be any return of the bleeding, and not only should this cease, but all vaginal discharges should be abolished. If bleeding does occur, one of two diseases is likely to be met with, either malignancy or fibroma. The importance of watching a woman through this period of her life cannot be too emphatically insisted upon. It is too generally the practice for physicians to attribute all the ills and complaints of such a patient to the menopause. If unusual symptoms do appear they must be carefully studied, and their cause, if possible, discovered, and if any pathological condition is discovered, then it must be treated as it would at any other period of a woman's life. To attribute vomiting to the nervous aspect of the change of life when an incipient carcinoma of the stomach is present would be a most unfortunate exhibition of diagnostic carelessness. It is frequently observed in highly neurotic women, where there is an hereditary taint of insanity, that this disorder may now develop. Paroxysmal tachycardia is often seen, too, during the climacteric, giving rise to most serious symptoms for the time; this is not due to any organic disease of the heart, but to local congestion of the heart-centre in the medulla oblongata, probably a reflex in most cases from the alimentary tract.

The depressing emotions have a bad influence on a woman at this period of life, hence worry, care, and anxiety and unnecessary responsibilities should be removed as much as possible. The flushes, tremblings, headaches, which are dependent on local congestion of certain areas of the nervous centres, are best relieved by the bromides. These remove the congestion and cause more or less a numbness of the areas. The potassium bromide is much slower and more permanent in its effect than the evanescent ammonium bromide, but in large doses its depressant effect on the heart is very objectionable. Can one imagine a more pitiable picture than a woman suffering from severe nervous manifestations of the climacteric accentuated by an induced cardiac depression? The best bromide of all to use is the sodium bromide, as it does not materially depress the heart, and it is also a diuretic. The use of opium and chloral should be absolutely forbidden, except for very short periods under the direct supervision of the medical attendant. The fear is that, at this impressionable time of a woman's life, one may induce the opium or chloral habit. There is no specific treatment of the climacteric, but each symptom must be combated as it is met with and treated secundum artem.

